# Epidemiological investigation and pathogenicity of porcine reproductive and respiratory syndrome virus in Sichuan, China

**DOI:** 10.3389/fmicb.2023.1241354

**Published:** 2023-09-13

**Authors:** Dike Jiang, Teng Tu, You Zhou, Yanwei Li, Yan Luo, Xueping Yao, Zexiao Yang, Meishen Ren, Yin Wang

**Affiliations:** ^1^Key Laboratory of Animal Diseases and Human Health of Sichuan Province, Sichuan Agricultural University, Chengdu, China; ^2^College of Veterinary Medicine, Sichuan Agricultural University, Chengdu, China

**Keywords:** porcine reproductive and respiratory syndrome virus (PRRSV), ORF5, lineage 8, pathogenicity, epidemic situation

## Abstract

Porcine reproductive and respiratory syndrome virus type 2 (PRRSV-2) lineage 8 was first detected in mainland China in 2006 and has since rapidly spread to become the primary epidemic strain in the country. In this study, samples such as lung tissue, hilar lymph nodes, abortion fetuses, and blood were collected from large-scale pig farms across 11 prefecture-level cities in Sichuan province between 2019 and 2020 for antigen detection and PRRS virus isolation. The antigen detection results indicated that the positive rate of HP-PRRSV (JXA1-Like strain) was 44.74% (51/114), NADC30-Like PRRSV was 17.54% (20/114), and classical PRRSV (VR2332-Like strain) was 37.72% (43/114). The predominant strain was HP-PRRSV. Positive samples were further inoculated into Marc-145 cells for virus isolation and identification, leading to the isolation of a new JXA1-Like PRRSV strain named SCSN2020. The strain was characterized by RT-qPCR, indirect immunofluorescence assay (IFA), plaque purification, electron microscopy, and whole genome sequencing. The total length of the viral genome was determined to be approximately 15,374 bp. A comparison of the SCSN2020 genome with VR2332 revealed that both strains had the same discontinuous 30-amino acid deletion on the Nsp2 gene. ORF5 genotyping classified the SCSN2020 strain as sublineage 8.7, with a whole genome sequence identity of 99.34% with JXA1. Furthermore, we evaluated the pathogenicity of the SCSN2020 strain in 28-day-old piglets and observed persistent fever from day 4 to day 10, weight loss started on day 7, dyspnea and severe lung lesions began started on day 14. The results of this study highlight the current PRRSV epidemic situation in Sichuan province and provide a scientific reference for subsequent prevention and control measures.

## 1. Introduction

Porcine reproductive and respiratory syndrome virus (PRRSV) is a major pathogen responsible for respiratory and reproductive diseases in pigs, significantly impacting the global pig industry. PRRSV is a single-stranded, positive-sense RNA virus that belongs to the *Arteriviridae* family (Mateu and Diaz, 2008; Guo et al., 2018). The genome size of PRRSV ranges between 15 and 15.5 Kb, encoding approximately 10 open reading frames (ORFs), including ORF1a, ORF1b, ORF2a, ORF2b, ORF3, ORF4, ORF5a, ORF5, ORF6, and ORF7 (Johnson et al., 2011; Fang et al., 2012). ORF1a and ORF1b constitute about 75% of the genome and encode at least 16 non-structural proteins (Nsps), including Nsp1a, NSPSP1β, Nsp2, Nsp2TF, Nsp2N, Nsp3-Nsp6, Nsp7α, Nsp7β, and NSP8-NSP12 (van Dinten et al., 1999; Firth et al., 2011). Structural proteins are encoded by ORF2-7 located at the 3′ end of the genome, including six membrane-associated structural proteins—GP2, E, GP3, GP4, GP5, and M—as well as nucleocapsid proteins (N proteins; Meng et al., 1995; Kappes and Faaberg, 2015; Lunney et al., 2016). Of these, ORF5 encodes the surface glycoprotein GP5 of virus particles, which plays a vital role in virus attachment, endocytosis, assembly, and the production of neutralizing antibodies (Mardassi et al., 1996; Dokland, 2010; Hicks et al., 2018). Additionally, the ORF5 gene has been widely used to investigate the molecular prevalence of PRRS and strain lineage classification (Shi et al., 2010; Gao et al., 2017; Guo et al., 2018).

Porcine reproductive and respiratory syndrome virus (PRRSV) has two primary genotypes—PRRSV-1 (European type, Lelystad Virus) and PRRSV-2 (North American type, VR2332). In China, PRRSV-2 has been the dominant genotype since its first identification in 1996 (An et al., 2007; Kuhn et al., 2016). Based on the ORF5 gene, PRRSV can be further categorized into nine lineages, with the most common lineages in China being lineage 1 (NADC30-Like), lineage 3 (QYYZ-Like), lineage 5 (VR2332-Like), lineage 8 (CH-1R and JXA1), and lineage 9. In 1996, sublineage 5.1, or VR2332-Like PRRSV, emerged in China but did not cause a pandemic (Li et al., 2007; Tian et al., 2007; Liu et al., 2021). However, sublineage 8.7, commonly known as highly pathogenic PRRSV (HP-PRRSV) JXA1, emerged in 2006 with a high fever as the main pathogenic feature, resulting in significant losses to the pig industry. QYYZ-Like PRRSV was initially identified in Guangdong, China, in 2010 and had a low sequence identity with other Chinese lineage strains, initially classifying it as a new lineage (Lu et al., 2015; Zhou et al., 2019). Sublineage 1.8, also known as NADC30-Like PRRSV, first appeared in China in 2012 (Zhao et al., 2015; Zhou et al., 2015) and is currently the most prevalent strain in the country. Another rapidly spreading strain in China is sublineage 1.5 or NADC34-Like PRRSV, first reported in 2017 (Bao and Li, 2021; Zhao et al., 2022a). The NADC34-Like strains accounted for 11.5 and 28.6% of positive cases in 2020 and 2021, and have spread to eight provinces in China (Xu et al., 2022). However, changes in lineage, gene recombination, and mutation of epidemic strains pose significant challenges to the preventing and controlling in China (Wang H. M. et al., 2018; Yu et al., 2022). Studies have shown that sublineage 8.7 (JXA1-like) and sublineage 1.8 (NADC30-like) have become the dominant strains prevalent in China’s swine herds, and NADC30-like strains have been highly regarded in recent years because of their high recombination and mutation rates. Multiple novel recombinant PRRSV strains from NADC30-Like outbreaks in China have recently been reported and exhibit different pathogenicity (Zhao et al., 2015; Liu J. K. et al., 2017; Chen et al., 2018). Additionally, the recombination of Chinese field strains and vaccine strains are emerging. These recombinant viruses exhibit significantly higher virulence than vaccine strains (Liu J. et al., 2017; Wang X. X. et al., 2018).

In this study, lung tissue, hilar lymph nodes, abortion fetuses, blood, and other pathogenic materials suspected of PRRSV infection were collected from large-scale pig farms in 11 prefecture-level cities in Sichuan province between January 2019 and January 2020 for pathogen detection and virus isolation. The highest positive rate was for HP-PRRSV, accounting for 44.74% (51/114) of samples. One strain of PRRSV was successfully isolated from the positive samples. Whole-genome sequencing and phylogenetic analysis classified the isolated strain as lineage 8 of PRRSV-2 (JXA1). Additionally, the pathogenicity of the isolated strain was evaluated through testing on 28-day-old piglets to provide scientific reference for subsequent prevention and control measures against PRRS in Sichuan province.

## 2. Materials and methods

### 2.1. Sample treatment

Clinical samples (including lung, hilar lymph node, abortion fetus, and blood) with suspected PRRSV infection were chopped up, freeze-thawed repeatedly, and then ground. After grinding was repeated three times, 2 mL PBS was added, centrifuged at 9710 × g for 5 min (4°C), and stored at −80°C. All clinical samples were collected from large-scale pig farms in Sichuan Province from 2019 to 2020.

### 2.2. Primer design and PRRSV detection in clinical samples

RNA was extracted using AG RNAex Pro RNA extraction reagent, and reverse transcription was performed using Evo M-MLV RT Kit (AG, Hunan, China). The reverse transcription reaction conditions were as follows: 37°C for 15 min, 85°C for 5 s, and 4°C ∞; the RNA reverse transcriptional reaction system is shown in Supplementary Table S1. Then the samples were detected by RT-qPCR and RT-PCR to confirm PRRSV positive. All primers are shown in Supplementary Table S2 (General Administration of Quality Supervision, Inspection and Quarantine of the People's Republic of China, Standardization Administration of China, 2018; Liao, 2019; Xu et al., 2019).

### 2.3. Isolation and identification of PRRSV

RT-qPCR-positive samples, including serum and lung tissue samples, were filtered through a 0.22 μm filter membrane to eliminate bacteria. For each sample, 1 mL of filtered supernatant was mixed with 1 mL of serum-free DMEM and added onto a monolayer of Marc-145 cells at a cell density between 80 and 90%. As described previously (Liu et al., 2022), a specific PRRSV polyclonal antibody labeled with fluorescein isothiocyanate (FITC) against PRRSV N protein (Bioss, Beijing, China) was used to detect PRRSV. As previously mentioned (Zhou et al., 2018a,b,c,d), the virus was purified by plaque analysis, and the virus titer was calculated according to the Reed-Muench method (Reed and Muench, 1938). Then, purified virus isolates were used to sequence their entire genomes. Meanwhile, to investigate the effect of different multiplicities of infection (MOI) on the growth of PRRSV in Marc-145 cells, we infected cells with MOI = 0.01 and MOI = 1 in triplicate. We incubated them for 1.5 h at 37°C. DMEM medium containing 2% serum was added for maintenance, and virus fluid was collected every 24 h and frozen at −80°C. After 5 days, we determined the virus titer and drew a growth curve. The PRRSV strain isolated from Sichuan province in 2020 was designated as SCSN2020.

### 2.4. Observation of virus particles using electron microscopy

The PRRS virus suspension was dispatched to Wuhan Servicebio Biotechnology Company for transmission electron microscopy analysis. We loaded 20 μL of the virus suspension onto a copper grid with a carbon film using a pipet gun, allowing it to incubate for 3–5 min. Excess liquid was carefully absorbed using filter paper. Subsequently, we applied 2% phosphotungstic acid to the copper grid, staining it for 1–2 min. Excess liquid was removed using filter paper, and the grid was left to dry at room temperature. Finally, the copper grids were observed under a transmission electron microscope (TEM), and images were captured.

### 2.5. Whole genome sequencing and bioinformatics analysis of PRRSV isolate

PRRSV RNA was extracted, and cDNA was prepared according to the above procedures and then sent to Beijing Tsingke Biotech Company for whole genome sequencing. Mega 6 (Tempe, AZ, United States) was used to analyze the whole genome homology and amino acid sequence of the primary genes and related genes of PRRSV strains in each region and the isolated strains in this study in the NCBI database, and the genetic evolution tree was constructed. The SeqMan program of DNAstar software version 7.0 (DNASTAR Inc., Madison, United States) was used to assemble the full-length genome sequence of the isolated strain. The PRRSV genome, ORF, and derived protein sequences were analyzed using DNAstar’s EditSeq and MegAlign programs.

### 2.6. Pathogenic experiment

Ten weaned piglets (about 28 days of age) were purchased from Chengdu Wangjiang Agriculture and Animal Husbandry Technology Company. All of them were negative for PRRSV antigen and antibody by RT-qPCR and ELISA assay. All the animals were randomly divided into two groups and separated into different rooms. All animals were individually housed under controlled temperature (26°C), humidity (60%), and lighting (12 h/day), with free access to water. Grouping of experimental animals: Ten weanling piglets were divided into the following groups: Group 1 (challenge group): Five weanling piglets were inoculated with 2 mL of virus solution (TCID50 = 10^5^/mL) via nasal drip; Five piglets in group 2 (control group) were inoculated with 2 mL DMEM/mL via an intranasal drop.

Following the viral challenge, clinical indicators were regularly monitored on the weanling piglets, including daily recordings of rectal temperature and observations of various clinical manifestations such as appetite, mental state, and breathing. The scores of the resulting clinical signs were recorded and tabulated in Supplementary Table S3 (Sun, 2017). Additionally, piglet weights were measured and documented on days 3, 7, 10, and 14 post-challenge to track any changes in growth patterns. These comprehensive evaluations aimed to assess the viral infection’s impact on the piglets’ health and welfare and provide insights into potential treatment options for this particular virus strain.

Viral load measurement of the weanling piglets was conducted by collecting blood, nasal, and oral secretions at regular intervals after the viral challenge (0, 3, 7, 10, and 14 days). RT-qPCR assays were used to detect signs of detoxification in these samples. Additionally, viremia levels in serum were assessed using the TCID50 method. These evaluations aimed to provide insights into the piglets’ viral clearance and detoxification mechanisms, thereby viral contributing to our understanding of how this particular virus strain interacts with its host.

If piglets died during the experiment, they were dissected immediately, and the gross pathological changes of each organ were observed. Lung and hilar lymph nodes were collected, rinsed with PBS to remove blood, soaked in 4% paraformaldehyde solution, fixed at room temperature for 24 h, and then embedded in the tissue, paraffin section and HE staining were used to observe the pathological changes. On day 14 of the challenge, the remaining pigs were euthanized and dissected, and tissues were collected and fixed as described above. Animal experiments in this study were approved by the Animal Ethics Committee of Sichuan Agricultural University (20220261). All experimental procedures and animal welfare standards strictly followed the guidelines of Animal Management at Sichuan Agricultural University.

### 2.7. Statistical analysis

Statistical analysis was performed with GraphPad Prism 8.0.2 (GraphPad Software, San Diego, CA, United States), and all data were using one-way ANOVA with the *t*-test. The level of significance was set at *p* < 0.05.

## 3. Results

### 3.1. RT-qPCR survey of clinical samples

Between 2019 and 2020, a total of 205 lung, hilar lymph nodes, serum, and abortion samples were collected in Sichuan to investigate the prevalence of PRRSV. Of these, 114 samples tested positive for PRRSV with an overall positivity rate of 55.61% (114/205). The highest positivity rate was observed in lung samples at 63.33% (38/60), as shown in Table 1. Specifically, 51 samples were positive for HP-PRRSV, 20 samples were positive for NADC30-like PRRSV, and 43 samples were positive for classical PRRSV; selected test results are presented in Supplementary Figure S1. In particular, the HP-PRRSV target band appeared at about 514 bp, while that of NADC30-like PRRSV appeared at approximately 334 bp (Supplementary Figure S1A). Additional RT-qPCR assays revealed that Supplementary Figures S1B–D corresponded to NADC30-like PRRSV, classical PRRSV, and HP-PRRSV, respectively.

**Table 1:** PRRSV detection results in each city of Sichuan Province from 2019 to 2020.

City	Lung (positive)	Hilar lymph nodes (positive)	Abortive fetal (positive)	Blood (positive)	Number of positive	Total sample number	Positive rate
Chengdu	4/7	2/5	–	2/6	8	18	44.44% (8/18)
Deyang	7/10	-	–	3/15	10	25	40% (10/25)
Guangyuan	2/3	1/1	2/3	3/5	8	12	66.67% (8/12)
Leshan	1/4	–	–	4/9	5	13	38.46% (5/13)
Meishan	2/3	–	–	7/10	9	13	69.23% (9/13)
Mianyang	1/2	0/1	1/2	3/6	5	11	45.45% (5/11)
Nanchong	1/2	–	–	3/9	3	11	27.27% (3/11)
Neijiang	3/5	–	–	7/10	10	15	66.67% (10/15)
Suining	15/20	3/5	9/16	18/25	45	66	68.18% (45/66)
Yaan	1/2	–	–	6/10	7	12	58.33% (7/12)
Yibin	1/2	–	–	3/7	4	9	44.44% (4/9)
Number of positives	38	6	12	58	114	–	–
Total sample	60	12	21	112	–	205	–
Positive rate	63.33% (38/60)	54.54% (6/11)	54.55% (12/22)	51.79% (58/112)	–	–	55.61% (114/205)

### 3.2. Isolation and identification of PRRSV

#### 3.2.1. Isolation and indirect immunofluorescence of PRRSV

**Figure 1:**
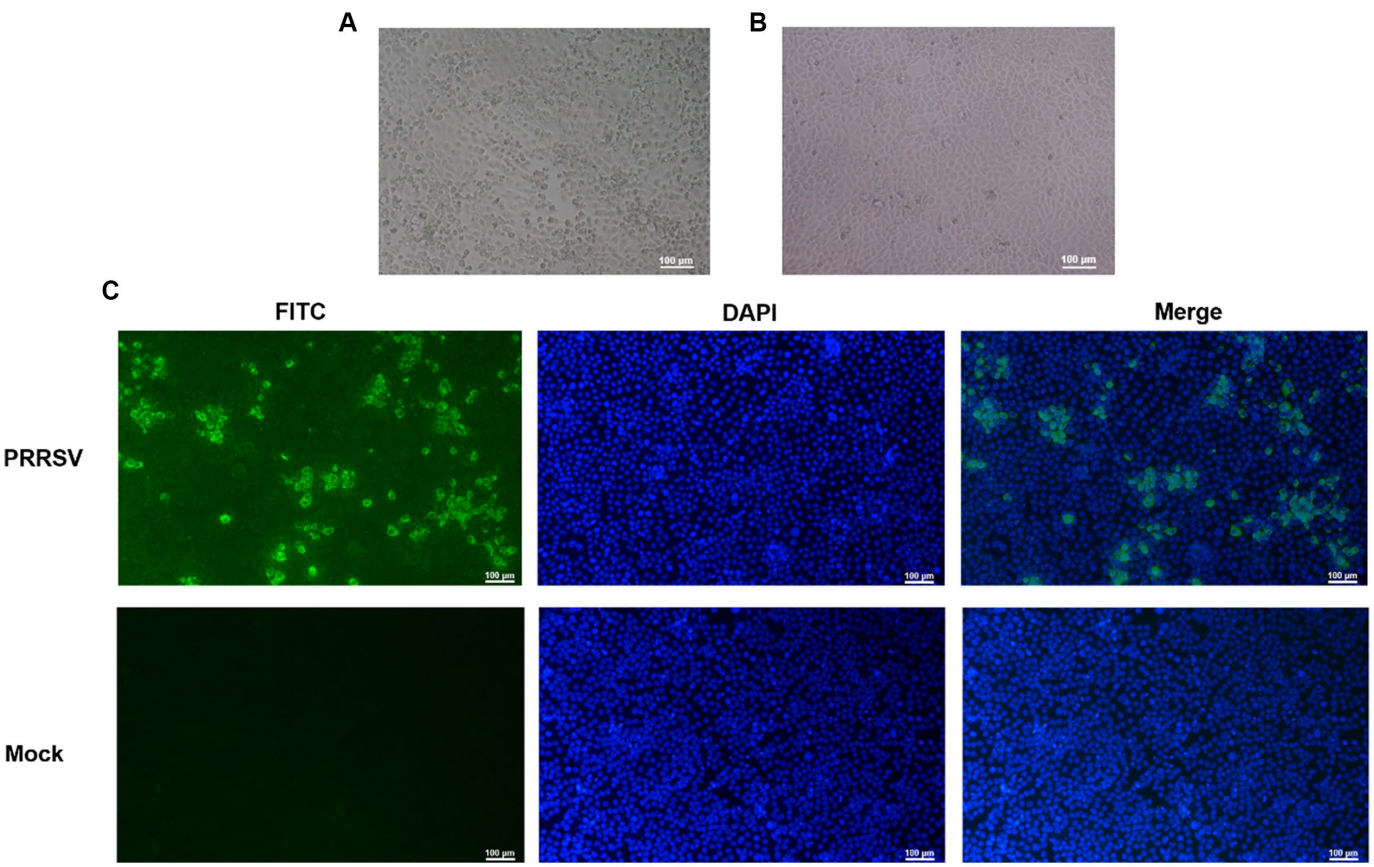
Identification of porcine reproductive and respiratory syndrome virus (PRRSV) isolates. **(A)** CPE diagram of Marc-145 cells infected with PRRSV isolates; **(B)** Blank control; **(C)** Results of indirect immunofluorescence assay.

The PRRSV-positive material was identified by RT-qPCR, which was inserted into Marc-145 cells and cultured in a 37°C CO2 incubator for 4–5 days. qPCR was performed every three passages. The results showed that one strain of PRRSV was isolated and cultured into Marc-145 cells for 48 h, the cells began to become larger and showed typical warp-like lesions. At 72 h, the lesions could reach 80%, showing mass or grape-shaped lesions (Figure 1A). The control group cells were in good condition with clear cell edges (Figure 1B).

To confirm infection with the PRRSV strain isolated from swine samples, indirect immunofluorescence was used to identify infected Marc-145 cells that had been cultured for 48 h. The cells were stained with rabbit anti-PRRSV N protein followed by FITC-labeled sheep anti-rabbit IgG, and the results are shown in Figure 1C. The staining pattern indicated that the isolated PRRSV strain could proliferate on Marc-145 cells, confirming our previous observations of morphological changes suggestive of viral infection.

#### 3.2.2. Results of TCID_50_ determination

To determine the infectious titer of PRRSV isolates, cell supernatant was collected, and performed a TCID_50_ assay using 96-well cell culture plates. The cell supernatant was diluted 10-fold and inoculated into the plates, which were then incubated at 37°C with CO2 for 4–5 days. We counted the number of cytopathic effects (CPEs) for each dilution and calculated the TCID_50_ of PRRSV using the Reed-Muench formula. The resulting titer was determined to be 10^–6.39/^0.1 mL (Supplementary Table S4).

#### 3.2.3. Plotting of viral one-step growth curves

To visualize virus growth over time, virus titers were plotted against corresponding time points by TCID_50_ assay to generate growth curves for each MOI (Supplementary Figure S2). Our results showed that when Marc-145 cells were infected with the MOI = 1, the virus titer reached its highest point on the second day, with a PRRSV titer of 10^–7.0^/mL. In contrast, when cells were infected with a MOI of 0.1, the virus titer reached its highest level of 10^–6.917^/mL on the third day. The TCID50 of PRRSV on the fifth day were 10^–5.833^/mL (MOI = 1) and 10^–5.917^/mL (MOI = 0.1), respectively.

#### 3.2.4. Results of virus plaque purification assay and transmission electron microscopy

**Figure 2:**
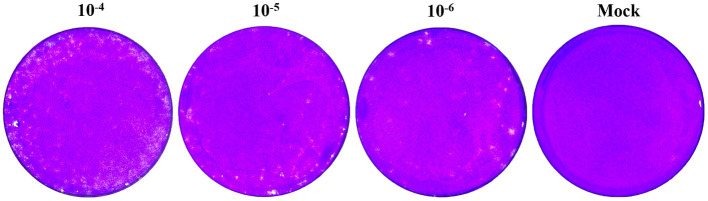
Plaque purification results.

A plaque assay and transmission electron microscopy analysis were performed to further characterize the isolated PRRSV strain. After 10-fold dilution of the virus solution, we inoculated 10^^−4^, 10^^−5^, and 10^^−6^ dilutions onto single-layer Marc-145 cells in six-well plate. After 5 days of culture in a 5% CO2 incubator at 37°C, uniform plaques appeared, displaying a typical PRRSV plaque pattern (Figure 2). The presence of these plaques confirms that the isolated PRRSV strain can infect and replicate within Marc-145 cells.

**Figure 3:**
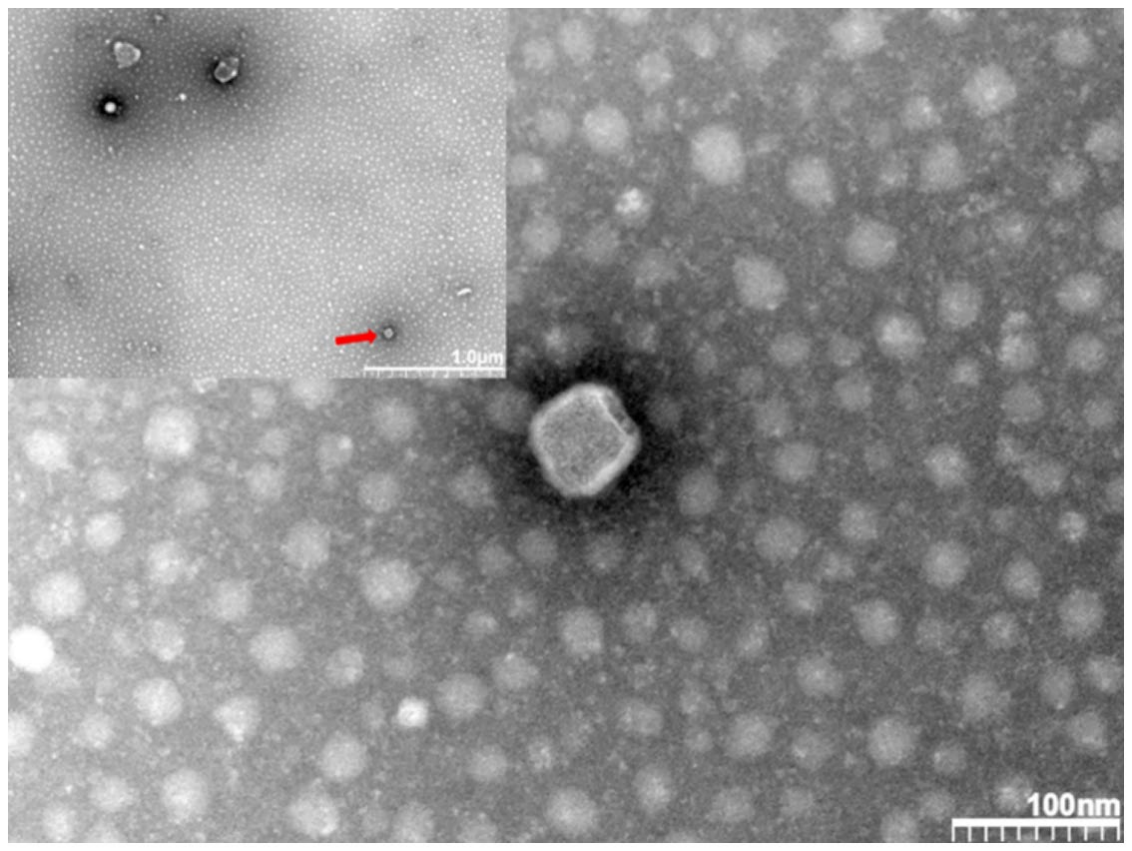
Transmission electron microscopic observation of virions.

In addition, we sent the virus solution to Wuhan Servicebio Biotechnology Company for transmission electron microscopy identification. Figure 3 shows polymorphic spherical virions of about 50–70 nm were observed in the supernatant, consistent with other observations of PRRSV virus particles by electron microscopy (Arteriviridae and Roniviridae, 2017). Based on these identification techniques, we designated this strain as PRRSV SCSN20.

### 3.3. Whole genome sequencing and bioinformatics analysis of PRRSV SCSN2020

#### 3.3.1. Whole genome sequencing and annotation of PRRSV SCSN2020

**Figure 4:**
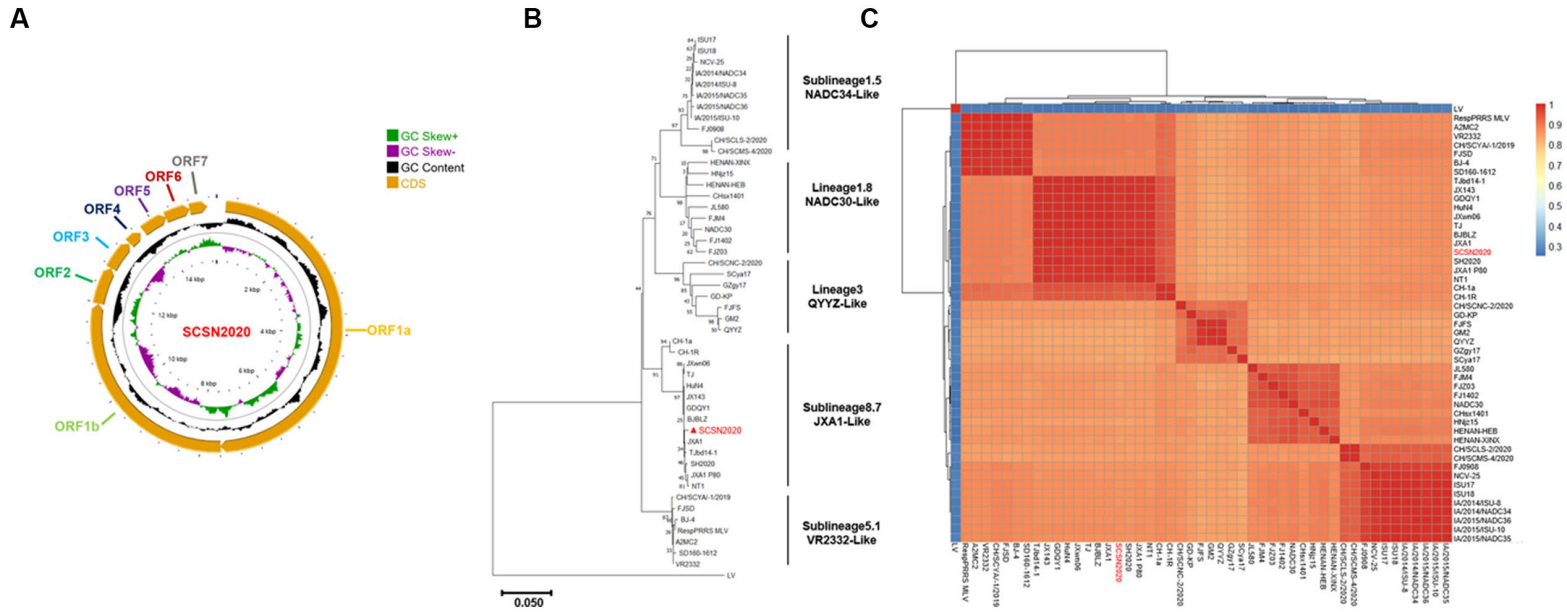
Whole genome sequencing and annotation of SCSN2020. SCSN2020 whole genome **(A)**, SCSN2020 phylogenetic tree based on PRRSV ORF5 gene **(B)**, cluster analysis of strains of each lineage of PRRSV **(C)**.

The sequencing results of PRRSV SCSN2020 were visualized using CG view. The full length of the PRRSV genome was 15,374 bp (GenBank accession number: OQ883907), with a GC content of 52.4%. ORF1a and ORF1b constituted 75% of the entire genome and were transcribed and translated into two polyproteins, pp1a, and pp1ab. pp1a was cleaved into Nsp1α, Nsp1β, Nsp2, Nsp3, Nsp4, Nsp5, Nsp6, Nsp7α, Nsp7β, and Nsp8, while pp1ab was cleaved into nonstructural proteins such as Nsp9, Nsp10, Nsp11, and Nsp12. Additionally, ORF2, ORF2b, ORF3, ORF4, ORF5a, ORF5, and ORF6 encode envelope proteins (E, GP2, GP3, GP4, GP5a, GP5, and M proteins) and N proteins, respectively (Figure 4A).

#### 3.3.2. Genetic evolution analysis of PRRSV SCSN2020

To assess the genetic relatedness between PRRSV SCSN2020 and other reference strains, we compared the nucleotide identity rates of the isolated strain with those of CH-1a, CH-1R, JXA1, NADC30, NADC34, VR2332, and QYYZ strains (Table 2). The results showed high levels of similarity between the isolated strain and most reference strains, with nucleotide identity rates ranging from 82.21 to 99.34%. Specifically, the sequence identity rate with LV strain was 58.87%, confirming that the isolated strain belonged to the PRRSV-2 strain. Further comparison with the sequences of PRRSV in the GenBank database revealed that the PRRSV SCSN2020 strain was closely related to the JXA1 strain. Specifically, the nucleotide and protein sequences of the 5’UTR, ORF1a (Nsp1α, Nsp1β, and Nsp2-8), and ORF1b (Nsp9-Nsp12) of PRRSV SCSN2020 were highly similar to those of JXA1, with nucleotide identity rates ranging from 97.39 to 100% and protein identity rates ranging from 95.51 to 100%.

**Table 2:** Sequence alignment results of PRRSV SCSN2020 with other reference strains.

Genome	CH-1A		CH-1R		JXA1		LV		NADC30		NADC34		VR2332		QYYZ	
	nt	aa	nt	aa	nt	aa	nt	aa	nt	aa	nt	aa	nt	aa	nt	aa
Complete genome	94.42		94.25		99.34		58.87		82.56		82.21		88.92		86.49	
5' UTR	97.37		95.79		100		54.05		91.62		92.59		91.58		95.26	
NSP1	94.34	92.43	94.26	92.43	99.04	99.22	55.33	48.07	84.86	85.9	84.25	86.42	89.03	90.08	88.42	88.51
NSP2	89.42	84.9	89.52	84.59	99.72	99.47	48.35	26.63	66.65	58.96	67.99	58.78	80.92	74.49	78.02	71.56
NSP3	95.07	98.43	95.07	98.43	99.63	99.55	58.92	57.81	83.41	91.03	82.88	91.03	90.88	98.43	82.06	88.57
NSP4	95.59	96.57	95.75	97.06	100	100	60.78	61.76	85.13	93.14	86.27	94.61	90.03	96.57	86.64	92.16
NSP5	94.51	95.29	93.92	94.71	100	100	63.8	71.76	90	94.12	84.34	88.89	89.61	95.29	81.96	90
NSP6	97.92	100	97.92	100	100	100	68.75	75	93.75	93.75	87.5	87.5	95.83	93.75	97.92	100
NSP7	96.01	96.91	95.62	96.53	99.23	100	52.48	40.51	82.63	84.94	82.11	86.87	89.45	89.58	93.82	94.21
NSP8	98.55	100	97.08	100	98.54	100	63.77	68.89	87.59	95.56	89.78	93.33	94.33	100	94.93	100
NSP9	96.46	98.59	96.41	98.44	99.06	98.91	66.74	73.02	87.19	97.03	87.24	96.88	92.29	97.66	91.25	97.34
NSP10	95.15	97.51	95.08	97.73	99.17	99.09	60.38	63.72	85.71	95.01	85.91	95.01	90.08	96.36	90.23	97.27
NSP11	94.79	97.77	95.09	98.66	98.81	99.11	66.52	76.44	91.07	96.43	86.31	96.43	90.48	95.54	88.39	94.64
NSP12	96.31	96.73	95.88	96.73	99.78	99.35	48.2	29.49	90.26	96.73	83.12	90.85	89.61	94.77	87.01	95.42
ORF2	95.86	95.31	95.34	94.92	98.84	98.83	63.31	59.38	85.77	85.55	86.8	85.16	92.63	91.8	89.26	88.28
ORF3	95.29	92.13	95.03	92.52	98.56	97.24	62.17	52.63	83.01	80.31	83.27	81.89	88.89	86.22	90.07	87.01
ORF4	96.65	98.31	96.46	97.75	97.39	95.51	65.04	67.76	86.96	89.89	86.22	90.45	89.76	91.01	94.23	95.51
ORF5	92.99	92.5	92.33	91	97.55	99	62.68	56.59	84.01	85.5	85.32	86.5	87.6	87.5	82.22	82
ORF6	97.33	97.7	96.95	97.13	99.81	100	69.14	78.74	88.76	93.1	88.95	93.68	95.24	97.7	90.66	96.55
ORF7	95.97	95.12	95.97	95.93	99.73	100	62.06	54.96	90.86	91.06	89.52	91.06	93.82	95.12	89.52	91.87
3' UTR	92.7		91.01		100		53.37		75.84		75.28		87.37		82.02	

To establish the genetic relationship between PRRSV SCSN2020 and representative strains of other PRRSV lineages, a phylogenetic tree was constructed based on the ORF5 gene sequence. The results showed that the other strains could be divided into four lineages based on the ORF5 gene except for the LV strain. Lineage 8 was mainly JXA1-Like, CH-1a-Like, TJ-Like, etc. Lineage 1 was mainly NADC30-Like and NADC34-Like. The third lineage was QYYZ-Like. Lineage 5 is VR2332-Like. The SCSN2020 strain isolated in this study was classified as lineage 8.7 (Figures 4B,C).

Compared with the VR2332-Like strain, the NSP2 protein sequence of the SCSN2020 isolate was missing one aa at 481aa. 533-561aa, deletion of 29 aa; Consistent with JXA1-Like (Supplementary Figures S3A,B); there were two Hypervariable regions (HVR) in ORF5 (Supplementary Figure S3C), HVR1 aa 33–35, SCSN2020 was NNN; HVR2 57–60 was AQKF; In addition, SCSN2020 contained three transmembrane regions and two virulence sites (aa 13 and aa 151), and the primary amino acid was arginine (R).

### 3.4. Pathogenicity analysis of SN2020

#### 3.4.1. Results of body temperature and body weight of piglets after the challenge

**Figure 5:**
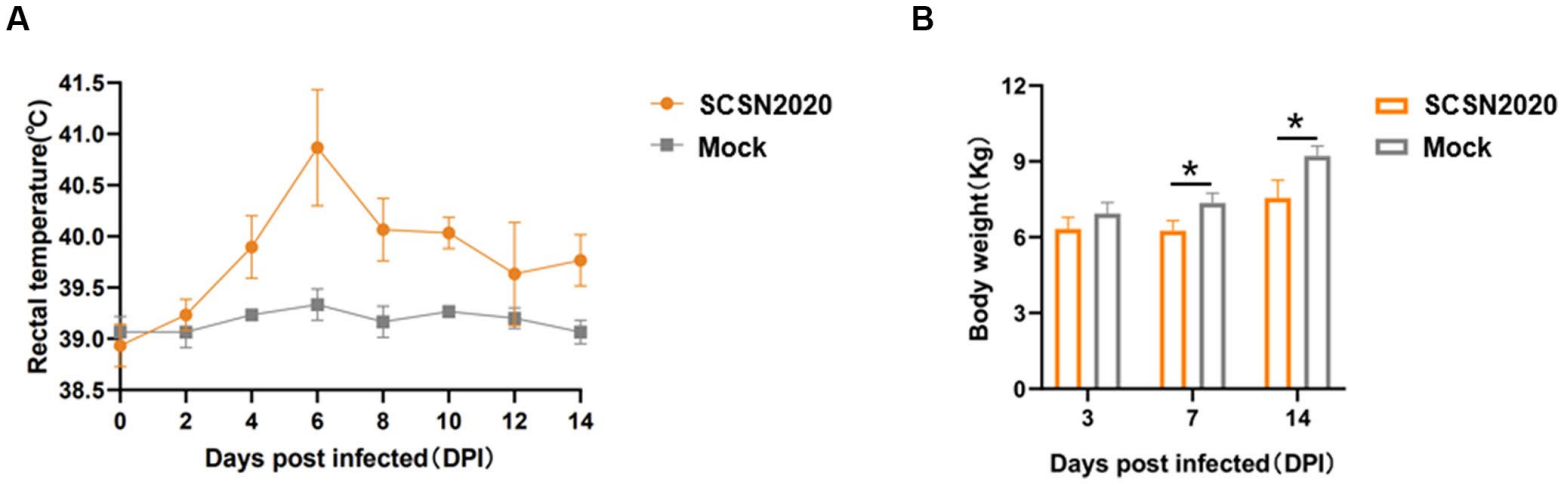
Changes in body temperature **(A)** and body weight **(B)** of piglets after challenge.

The results are presented in Figures 5A,B. In Figure 5A, it can be seen that the body temperature of the challenged piglets started to rise on the fourth day after the challenge. On the sixth day, it had increased to 40.65°C, and all the challenged pigs were above 40°C. From the 10th day onwards, the population temperature began to decrease. On the other hand, the control pigs showed no abnormalities throughout the experiment.

In Figure 5B, it can be observed that there was no significant difference between the control group and the challenge group for the first 3 days. However, from day 7 and day 14, the body weight of piglets in the challenge group was significantly lower than that in the control group (*p* < 0.05).

The body weight of piglets in the challenge group decreased by 1.10 and 1.66 kg, respectively. This indicates that the strain could decrease the weight growth of piglets, and the difference was more significant with the extension of time.

#### 3.4.2. Clinical symptom score results of piglets after challenge

**Figure 6:**
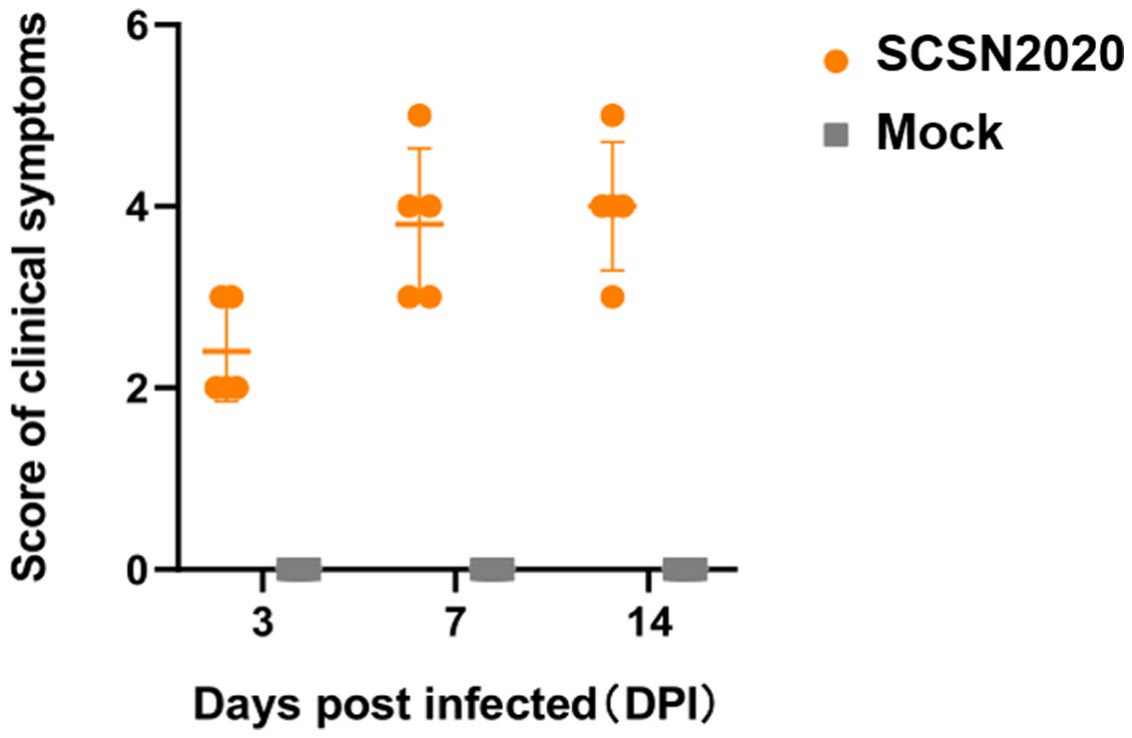
Changes in clinical symptom scores of piglets after the challenge.

The results are presented in Figure 6. On the challenge’s third day, pigs’ feed intake decreased, and some pigs showed clinical signs such as runny noses and clumps. On day 4, the clinical signs worsened, and the pigs developed a decreased appetite, eyelid edema, and an indirect mild cough. By the seventh day, the pigs began to cluster, sneeze, cough, depression, and have other clinical signs. A few pigs also showed clinical signs such as ear redness and abdominal breathing.

On day 14, one of the pigs had dyspnea, stopped feeding, and had obvious abdominal breathing. These clinical signs suggest that the strain had adverse effects on the respiratory system of the piglets. The decrease in feed intake and appetite also indicates that the strain had negative impacts on the digestive system of the piglets.

#### 3.4.3. Detection of detoxification of piglets after the challenge

**Figure 7:**
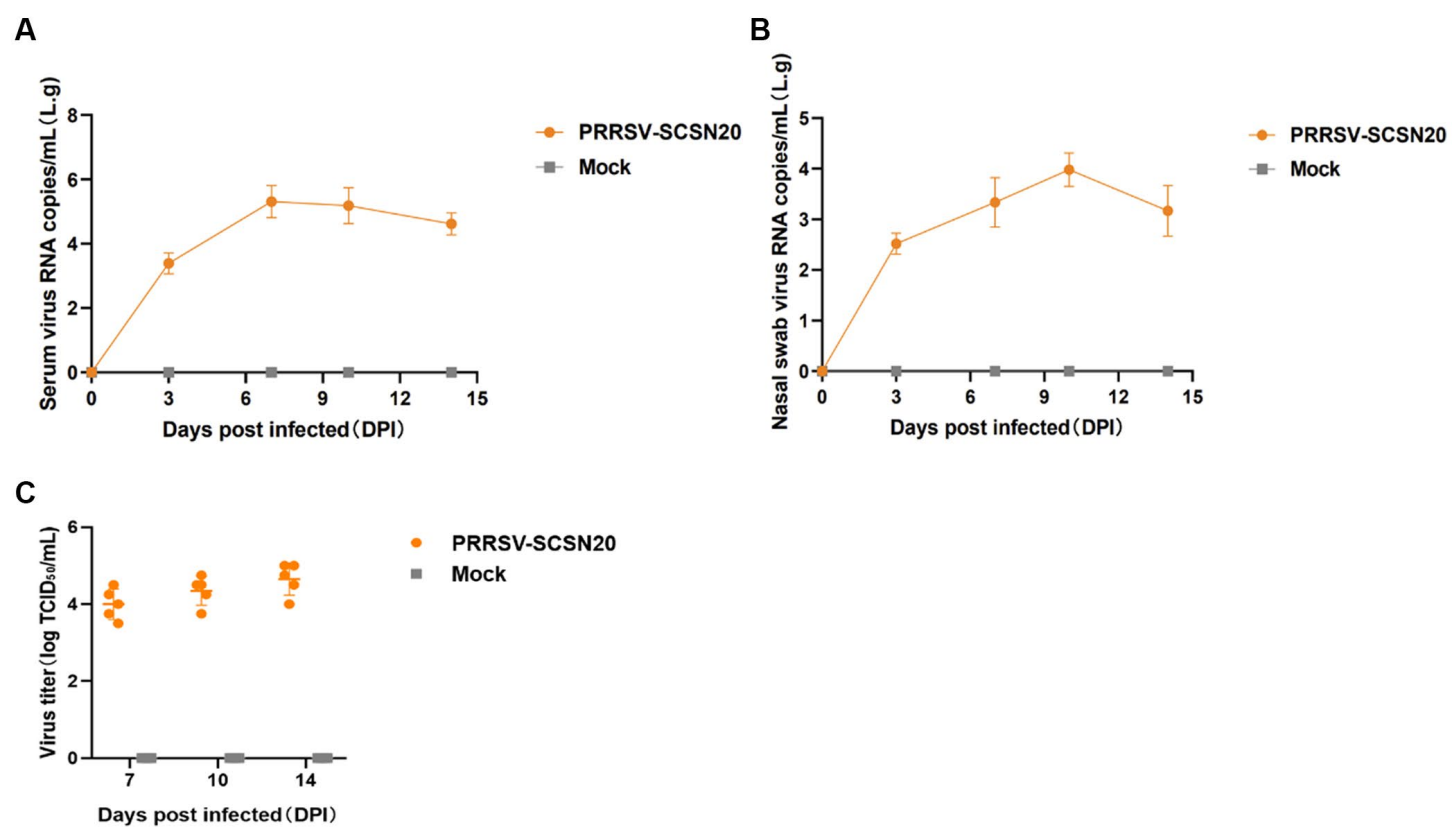
Shedding of virus and changes in blood virus titers in piglets after challenge. The amount of virus in blood in blood **(A)** and nasal swabs **(B)**, virus titer in the serum **(C)**.

The results are presented in Figure 7. On day 7 after the challenge, the amount of virus in blood reached the peak (2.05 × 10^5^ copies/mL) and then began to slowly decline (Figure 7A). Similarly, the amount of virus in nasal swabs peaked at 9.58 × 10^3^ copies per milliliter on day 10 after the challenge and then began to decline (Figure 7B). The virus titer in the serum of piglets was 10^4^ TCID50/ml on day 7, 10^4.35^ TCID50/mL on day 10, and 10^4.65^ TCID50/mL on day 14 after infection (Figure 7C).

#### 3.4.4. Pathological observation after dissection

**Figure 8:**
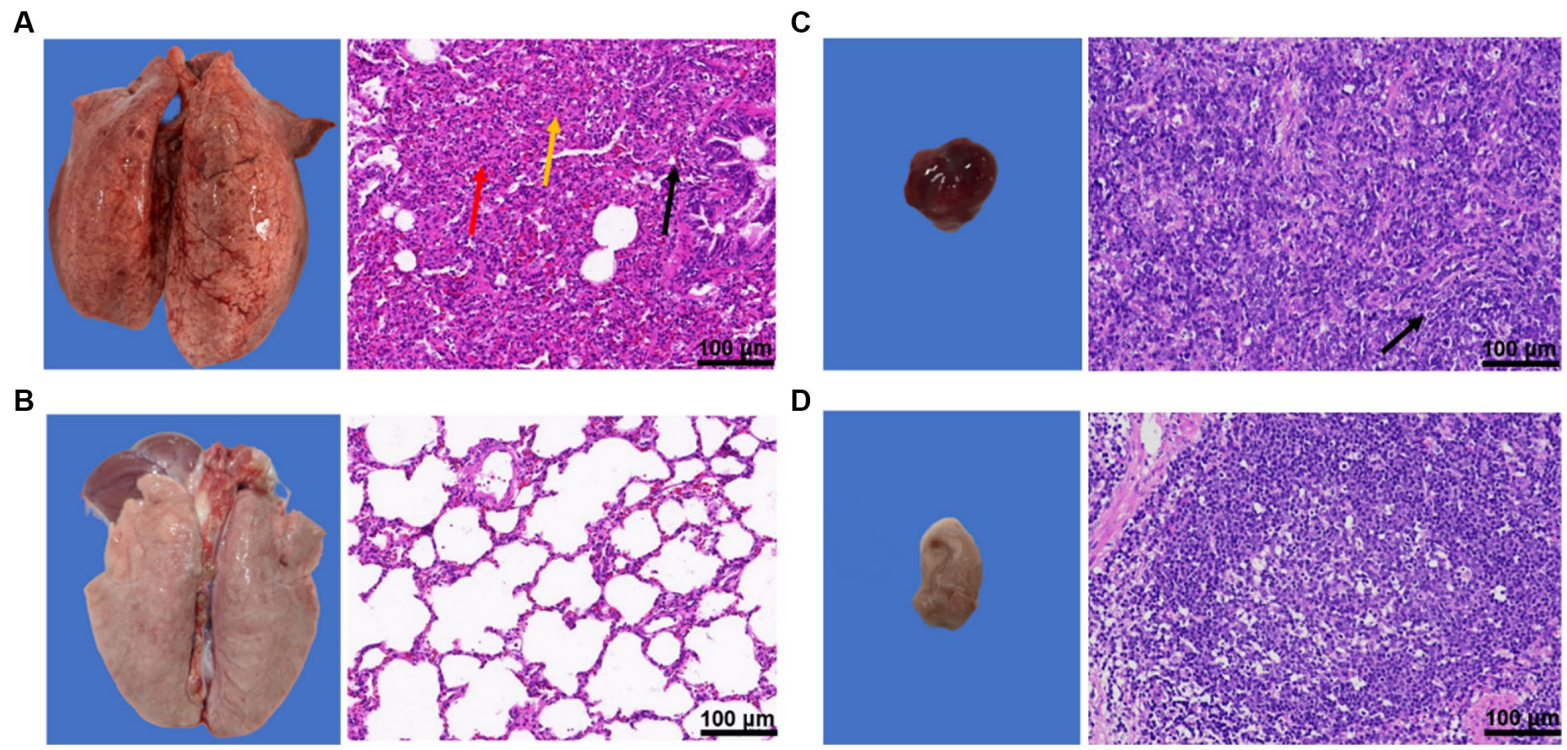
Results of pathological changes in lung and hilar lymph nodes of piglets after challenge. **(A)** lung and pathological sections of the challenge group; **(B)** Lung and pathological sections of the blank control group; **(C)** Lung lymph nodes and pathological sections of the challenge group; and **(D)** Lung lymph nodes and pathological sections of the blank control group.

The piglets in the challenge group showed obvious pneumonia, with tissue consolidation and multiple foci in the apical lobe of the lung, widening of the lung interstitium, and obvious bleeding points on the lung surface (Figure 8A). The hilar lymph nodes were enlarged and bleeding (Figure 8C). The results of pathological sections showed that the overall structure of the lung tissue of piglets in the challenge group was abnormal, with unclear alveolar tissue structure, significant collapse, thickening of the alveolar wall and parenchymal tissue, slight congestion of the lung interstitium, and significant infiltration of inflammatory cells (Figure 8A). The structure of pulmonary lymph nodes was not clearly defined, and the boundary between the cortex and medulla was unclear, with a large amount of diffuse lymphoid tissue visible (Figure 8C).

In contrast, the blank group showed normal lung tissue structure, with no alveolar fusion, expansion, atrophy, or other degeneration observed. There was no obvious loss, edema, or shedding of the alveolar epithelial cells in the tissue, no thickening of the alveolar wall, and no inflammatory cell infiltration. The lymphoid nodules were tightly packed with clear boundaries between the cortex and medulla of the tissue, and abundant numbers of lymphocytes (Figures 8B,D).

## 4. Discussion

Porcine Reproductive and Respiratory Syndrome (PRRS) first emerged in China in 1996, rapidly spreading across the country and causing significant economic losses to the pig industry (An et al., 2007; Sui et al., 2018). Currently, vaccination represents the main strategy for preventing and controlling PRRS outbreaks. However, a contentious issue has arisen regarding the effectiveness of PRRSV vaccines; in clinical settings, such vaccines are frequently inefficient and ineffective (Guo et al., 2018). Nevertheless, given the prevalence of PRRS, vaccination remains the primary approach available. Commercially available vaccines in China include CH-1a/CH-1R, R98/R98 MLV, VR2332/Ingelvac PRRS MLV, JXA1/JXA1-R, TJ/TJM-F92, HuN4/ Hun4-F112, GD/GDr180, and among others (Leng et al., 2017; Wang H. M. et al., 2018; Ding et al., 2021). While these vaccines can partially control PRRS outbreaks and reduce the duration of clinical signs and viremia in pigs, they cannot provide full protection against PRRS infection, with vaccine efficacy limited to partial or modest protection against heterologous challenges.

However, mass vaccination with attenuated vaccines can pose safety risks and lead to genetic variations (Wang et al., 2015; Zhou et al., 2017). Studies indicate that vaccinated pigs can transmit vaccine viruses to unvaccinated pigs, thus propagating different vaccine strains within pig populations (Li et al., 2016, 2017; Bian et al., 2017; Wang X. X. et al., 2018). Large-scale vaccination with attenuated vaccines may also contribute to the emergence and spread of PRRSV-2 (Shi et al., 2013). Furthermore, “reverse virulence” has been reported during attenuated vaccine administration (Jiang et al., 2015). The misuse and overuse of vaccines have also contributed to the emergence of complex strains of PRRSV, notably NADC30-Like PRRSV, which predominantly recombines with HP-PRRSV (JXA1), classical PRRSV (CH-1R, VR2332), and attenuated vaccines (TJM-F92) to produce novel strains (Wang et al., 2016). In addition to the recombination between vaccine strains and other PRRS viruses, HP-PRRSV strains in China demonstrate recombination patterns across distinct lineages and sublineages. HP-PRRSV strains emerged in 2006, represented by JXA1, TJ, and HuN4, also parental strains of JXA1-R, TJM-F92, and HUN4-F112. In a previous study, we found that since 2016, the prevailing strains in Sichuan Province are mainly VR2332-Like, JXA1-Like, and NADC30-Like, and present different ways of recombination, mainly as follows: JXA1-Like+NADC30 -Like (Zhou et al., 2018a,b,c,d); VR2332-Like+JXA1-Like+NADC30-Like (Zhou et al., 2018a,b,c,d); JXA1-Like+NADC30-Like+QYYZ-Like (Zhou et al., 2018a,b,c,d). These studies further demonstrate that recombination may occur between PRRSV strains and vaccine strains. Lineage 3 surfaced in 2010, typified by QYYZ and GM2, among others. Subline 1.8 emerged in 2013, exemplified by NAD30-Like (Zhang et al., 2017, 2018; Zhou et al., 2018a,b,c,d). This study found that the positive rates of HP-PRRSV and NADC30 like PRRSV antigens were 44.74% (51/114) and 17.54% (20/114), respectively. As a large province of domestic pig stocking and slaughtering, Sichuan Province has widely used vaccines in pig herds, which also facilitates the recombination of PRRSV strains. At the same time, it has also brought enormous pressure to prevent and control PRRSV epidemics.

In addition to recombination, mutation is a crucial mechanism in the evolution of PRRSV (Guo et al., 2018). The exchange of gene segments usually occurs between different circulating strains and confers new biological characteristics to other variants, mainly including changes in pathogenicity and antigenicity (Sun et al., 2016; Bian et al., 2017). The Nsp2 and ORF5 regions of the PRRSV genome are highly susceptible to mutation, which makes them ideal targets for monitoring the development of PRRS gene mutations. Notably, HP-PRRSV has a 30-amino-acid discontinuous deletion (1aa + 29aa) in the NSP2 coding region (Tian et al., 2007), whereas NADC30-Like PRRSV presents a 131-amino-acid discontinuous deletion (111aaa + 1a + 19aa; Brockmeier et al., 2012). Meanwhile, NADC34-Like exhibits a continuous 100-amino-acid deletion (Bao and Li, 2021). Sequence comparison in this study found that compared with the genome of the VR2332 strain, the isolate SCSN2020 had 30 amino acid discontinuous deletions (1aa and 29aa) in the NSP2 coding region, which was located at positions 481 and 533–561, respectively.

The hypervariable region (HVR) of the PRRSV GP5 protein also shows significant variations. For instance, in aa 33–35 of HVR1, HP-PRRSV predominantly features NNN, while NADC34-Like mainly exhibits NNS or SSS, NADC30-Like primarily shows SNS, and QYYZ-Like features GNS; by contrast, VR2332 is characterized by NDS. Concerning HVR2 in aa 57–60, HP-PRRSV mainly exhibits AQKF or ANKF, whereas NADC34-Like mainly displays NKSF, NADC30-Like is more variable, showing mainly NEKF, STKF, NKKF, among others, and QYYZ-Like mainly exemplifies ATNF or ANKF. VR2332 is mainly ANKF. In PRRSV GP5, amino acids 13 and 151 act as virulence determinants (Allende et al., 2000; Luo et al., 2023). This study found that SCSN2020 mainly features arginine (R), with the remaining lineages predominantly displaying lysine (K), glutamine (Q), isoleucine (I), and arginine (R).

This study analyzed 205 suspected PRRSV samples collected from 11 prefecture-level cities in Sichuan province between 2019 and 2020. Of the 114 PRRSV-positive samples, the antigen-positive rate of HP-PRRSV (JXA1-Like strain) was 44.74% (51/114), NADC30-Like PRRSV was 17.54% (20/114), and classical PRRSV (VR2332-Like strain) was 37.72% (43/114), indicating that HP-PRRSV remains the prevalent strain in Sichuan province. Notably, 20 samples of NADC30-Like PRRSV were detected, with other studies indicating an increasing detection rate of this strain in the region (Zhou et al., 2018a,b,c,d; Zhao et al., 2022b). Furthermore, recent recombinant strains of PRRS in Sichuan province have mainly featured NADC30-Like PRRSV, while minor strains included JXA1-Like and TJ-Like HP-PRRSV. Previous studies have shown that HP-PRRSV, including JXA1, TJ, and HuN4, are lethal to piglets. These viruses are characterized by high fever, high morbidity (50–100%), and high mortality (20–100%; Tong et al., 2007; Liu et al., 2022). New recombinant strains can also lead to changes in PRRSV virulence. For example, the TJnh1501 strain is a recombinant virus between NADC30-Like and MLV vaccine (TJMF92), and its virulence is between HP-PRRSV (JXwn06) and MLV strains Between; SCN17 strain is recombined from NADC30-Like, JXA1-Like and MLV vaccine (VR2332), causing persistent fever and moderate interstitial pneumonia in pigs (Zhou et al., 2018a,b,c,d); FJLIUY-2017 strain is lineage 1 (NADC30-Like), lineage 3 (QYYZ-Like), lineage 5.1 (VR2332-Like), and lineage 8.7 (JXA1-Like; Liu et al., 2019). SCSN2020 isolated in this study was identified as JXA1-Like. Although no recombination occurred, our pathogenicity study revealed that piglets in the challenge group presented with typical PRRS clinical symptoms signs and pathological changes, including continuous increases in body temperature, weight loss, and viremia, as well as hunger, severe shortness of breath, and labored breathing from day 13. These findings highlight the continued high pathogenicity of HP-PRRSV and underscore the need for vigilance in preventing and controlling its spread. Moreover, given Sichuan’s status as a major pig-raising province in China, where swine vaccines are widely used, the potential for recombination between HP-PRRSV and NADC30-Like PRRSV is heightened. Recombinant strains exert significant pressure on PRRS prevention and control efforts. Hence, our study provides a scientific reference for future efforts to regulate and manage PRRSV outbreaks in Sichuan province.

## 5. Conclusion

In summary, we counted the prevalence of PRRSV in Southwest China from 2019 to 2020 and found that the highest positive rate of pathogen detection is still HP-PRRSV, and through isolation, purification, and whole-genome sequencing analysis, a strain of HP-PRRSV (SCSN2020) was obtained. Pathogenicity experiments found that the strain had typical PRRSV symptoms and pathological changes. In addition, multiple recombinations occurred between sublineage 8.7 (JXA1-Like) and lineage 1.8 (NADC30-Like) prevalent in China, and our study also reminds the importance of monitoring PRRSV in China.

## Data availability statement

The datasets presented in this study can be found in online repositories. The names of the repository/repositories and accession number(s) can be found in the article/Supplementary material.

## Ethics statement

The animal study was approved by Sichuan Agricultural University Institutional Animal Care and Use Committee. The study was conducted in accordance with the local legislation and institutional requirements.

## Author contributions

DJ, MR, and TT: conceptualization. DJ: software. DJ and TT: methodology and writing—original draft preparation. YZ, MR, and Y Li: formal analysis. YZ: investigation. XY, Y Luo, and YW: resources. DJ and MR: data curation. DJ, ZY, and YW: writing—review and editing. DJ and YW: visualization. MR and YW: supervision. ZY, Y Luo, and XY: project administration. Y Luo and YW: funding acquisition. All authors contributed to the article and approved the submitted version.

## Funding

This project was supported by the Sichuan Province Science and Technology Planning Program (2021ZDZX0010 and 2021YFSY0005).

## Conflict of interest

The authors declare that the research was conducted in the absence of any commercial or financial relationships that could be construed as a potential conflict of interest.

## Publisher’s note

All claims expressed in this article are solely those of the authors and do not necessarily represent those of their affiliated organizations, or those of the publisher, the editors and the reviewers. Any product that may be evaluated in this article, or claim that may be made by its manufacturer, is not guaranteed or endorsed by the publisher.
