# Supplementary figures and images for: Epidemiological investigation and pathogenicity of porcine reproductive and respiratory syndrome virus in Sichuan, China
Source: Front Microbiol. 2023 Sep 13;14:1241354. doi: 10.3389/fmicb.2023.1241354 (PMC10533931; doi:10.3389/fmicb.2023.1241354)

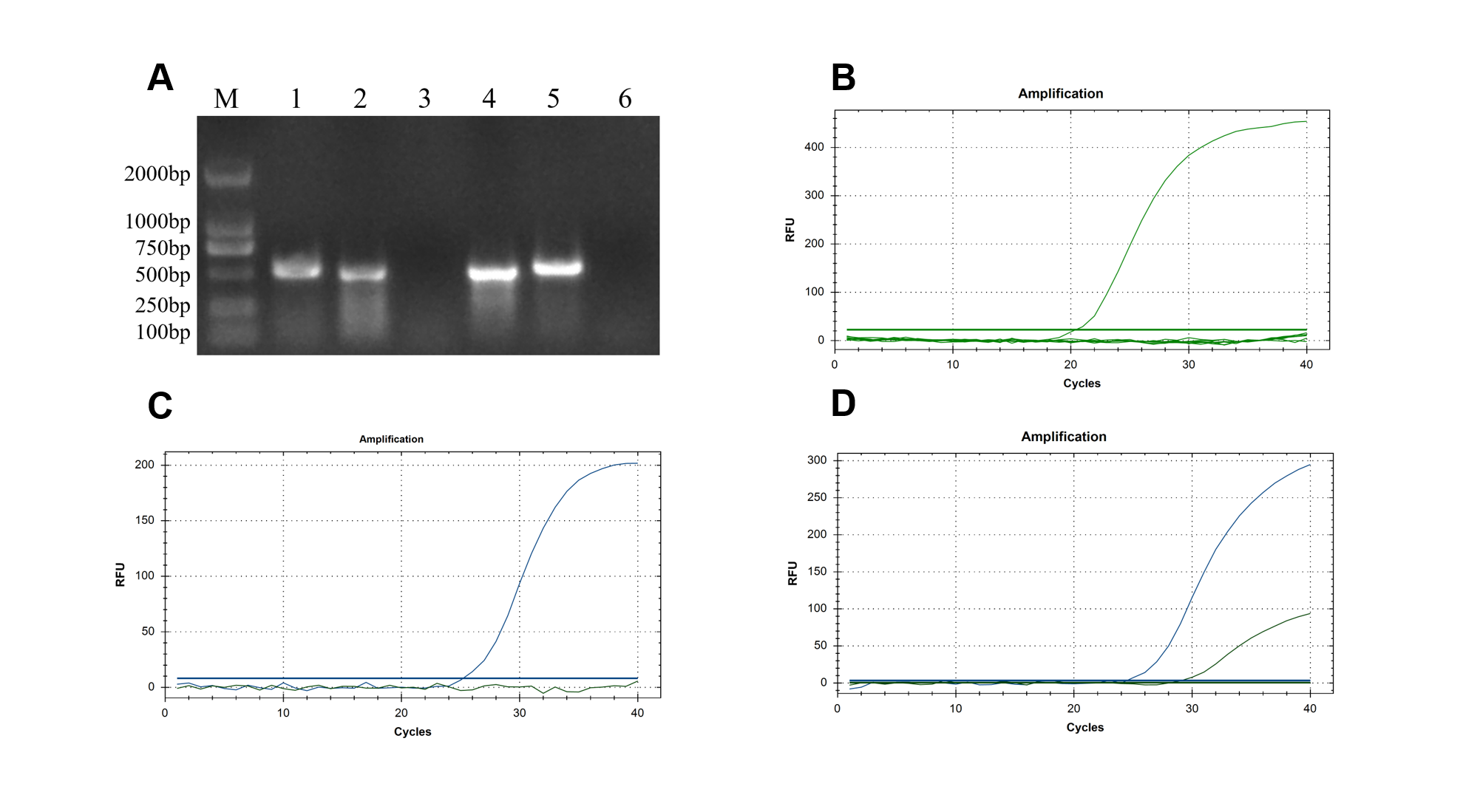

Supplement: Supplementary file 1 [file Data_Sheet_1.zip › Supplementary material/Figure/Supplementary Figure S1.tif]

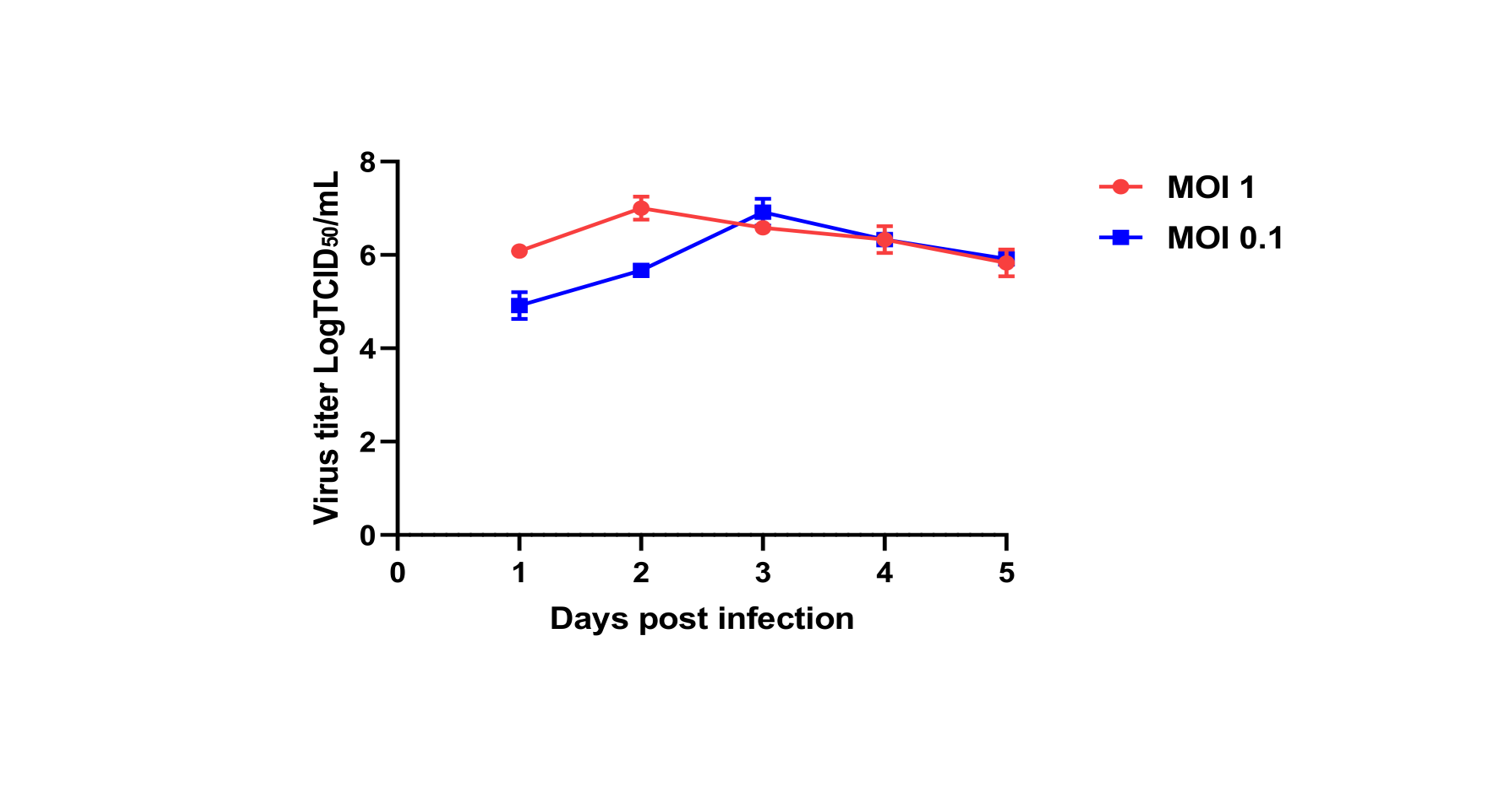

Supplement: Supplementary file 1 [file Data_Sheet_1.zip › Supplementary material/Figure/Supplementary Figure S2.tif]

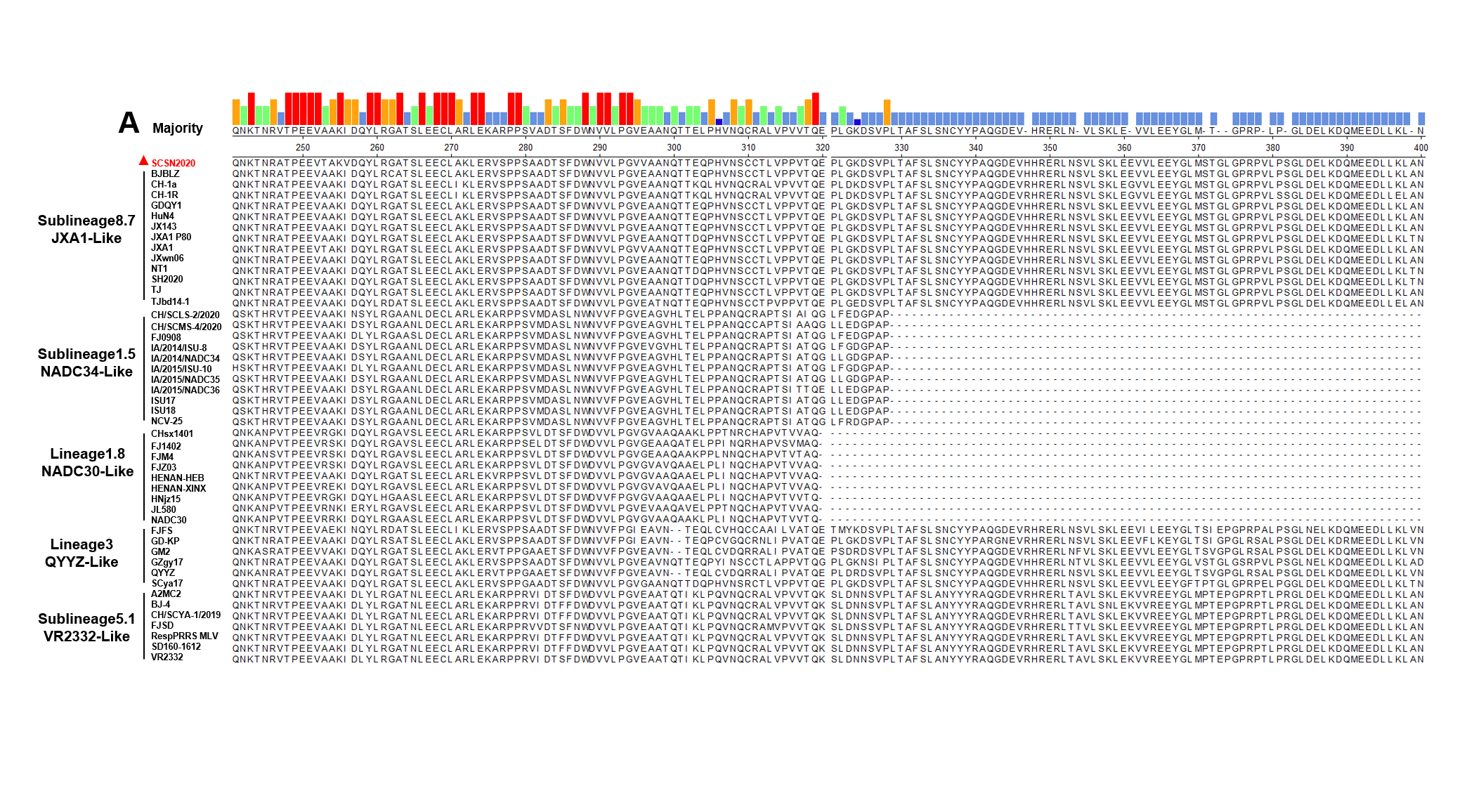

Supplement: Supplementary file 1 [file Data_Sheet_1.zip › Supplementary material/Figure/Supplementary Figure S3-A.tif]

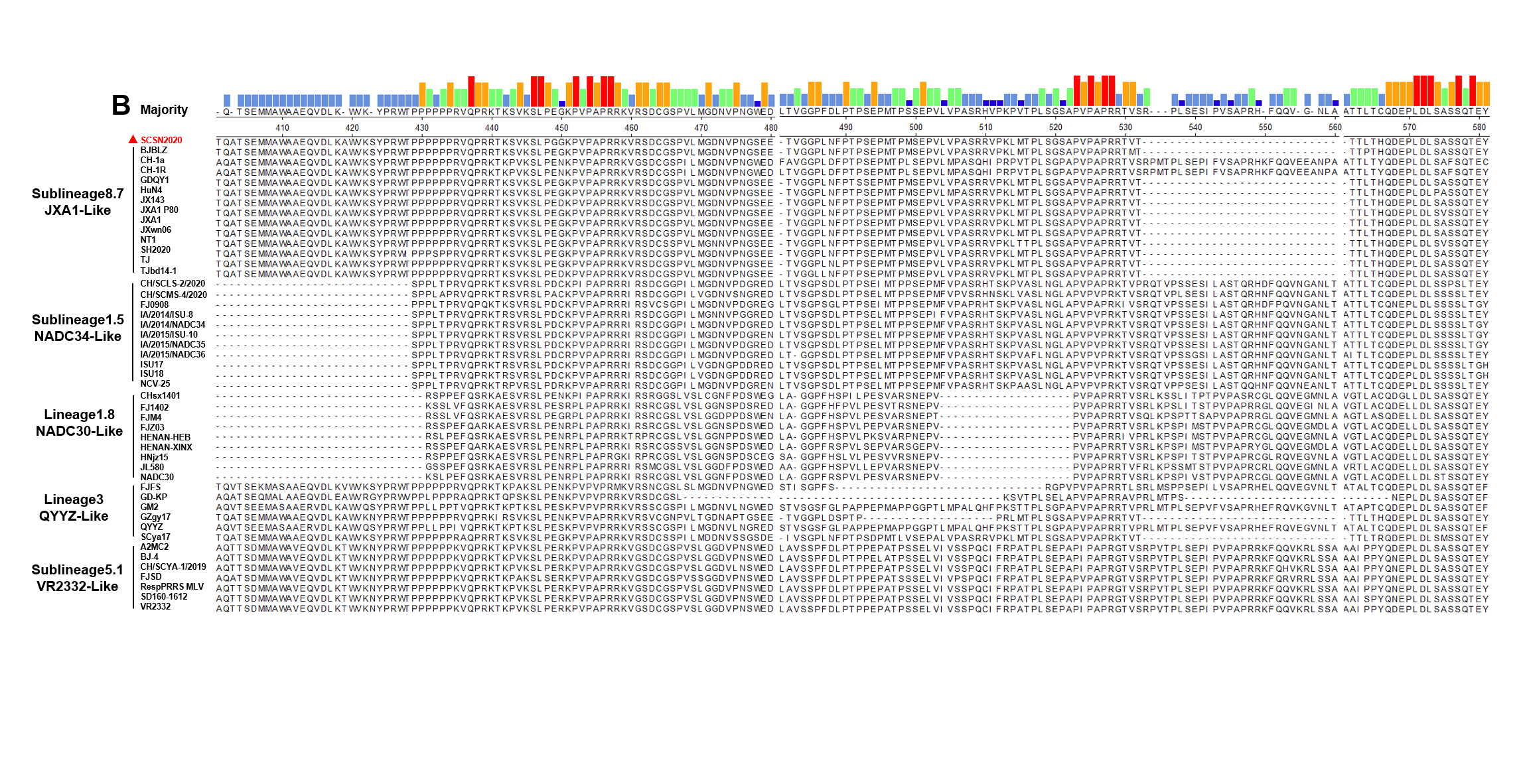

Supplement: Supplementary file 1 [file Data_Sheet_1.zip › Supplementary material/Figure/Supplementary Figure S3-B.tif]

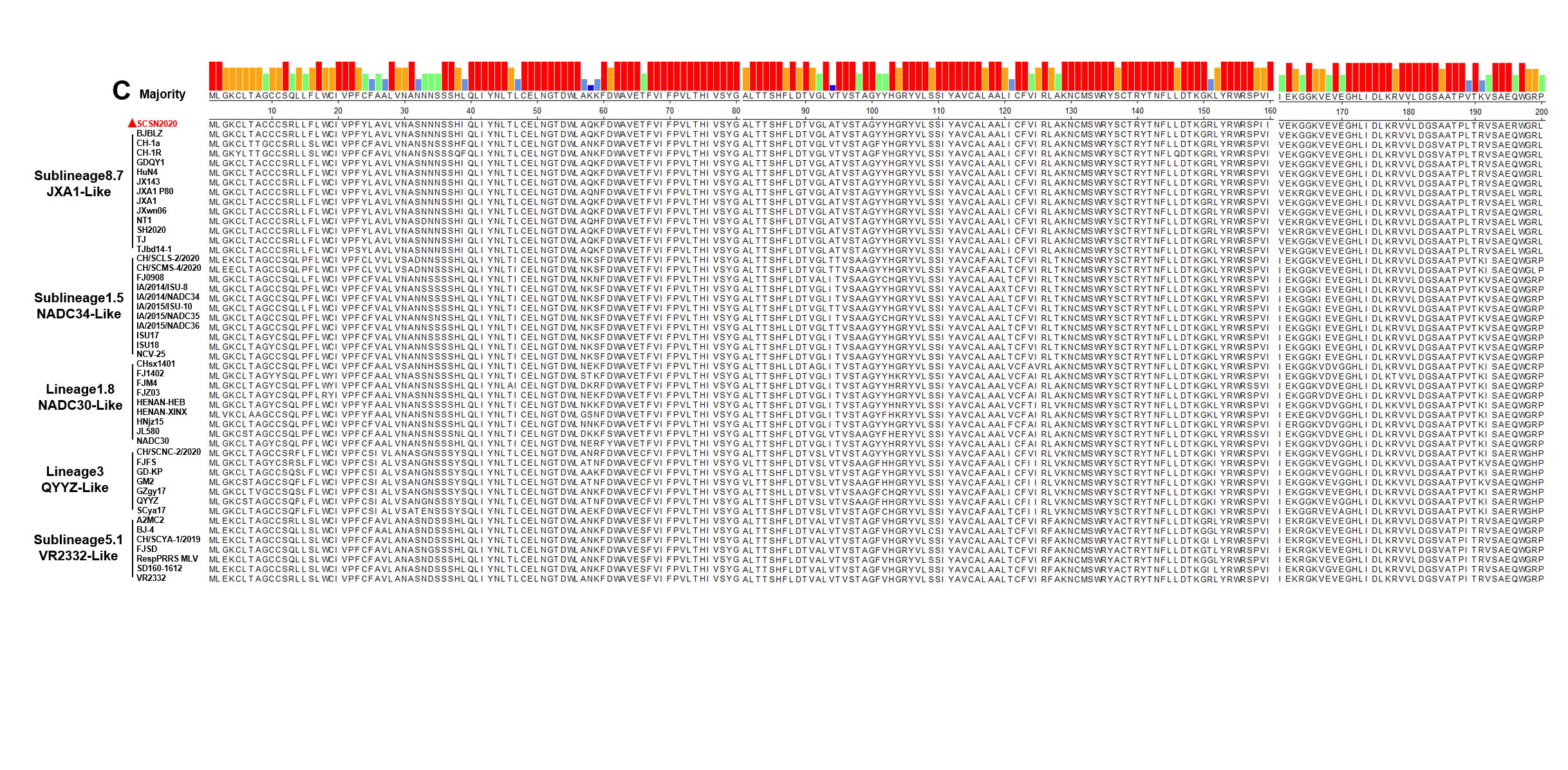

Supplement: Supplementary file 1 [file Data_Sheet_1.zip › Supplementary material/Figure/Supplementary Figure S3-C.tif]
